# Caveolin-2 deficiency induces a rapid anti-tumor immune response prior to regression of implanted murine lung carcinoma tumors

**DOI:** 10.1038/s41598-019-55368-4

**Published:** 2019-12-12

**Authors:** Yajun Liu, Xiaoqiang Qi, Guangfu Li, Grzegorz Sowa

**Affiliations:** 1 Department of Medical Pharmacology and Physiology, University of Missouri-Columbia, Columbia, MO 65212 USA; 2 Department of Surgery, University of Missouri-Columbia, Columbia, MO 65212 USA; 3 Ellis Fischel Cancer Center, University of Missouri-Columbia, Columbia, MO 65212 USA; 4 Department of Molecular Microbiology and Immunology, University of Missouri-Columbia, Columbia, MO 65212 USA

**Keywords:** Lung cancer, Tumour immunology

## Abstract

Immunosuppression is critical for tumor growth and metastasis as well as obstacle to effective immunotherapy. Here, we demonstrate that host deficiency in caveolin-2, a member of caveolin protein family, increases M1-polarized tumor-associated macrophage (TAM) and CD8 T cell infiltration into subcutaneously implanted murine lung carcinoma tumors. Importantly, increase in M1 TAM-specific markers and cytokines occurs prior to increased numbers of tumor-infiltrating CD8 T cells and tumor regression in caveolin-2 deficient mice, suggesting that an early increase in M1 TAMs is a novel mechanism, via which host deficiency in caveolin-2 inhibits tumor growth. Consistent with the latter, transfer and co-injection of caveolin-2 deficient bone marrow (origin of TAMs) suppresses tumor growth and increases numbers of M1-polarized TAMs in wild type mice. Collectively, our data suggest that lung cancer cells use caveolin-2 expressed in bone marrow-derived cell types including TAMs to promote tumor growth via suppressing the anti-tumor immune response and that caveolin-2 could be a potential target for cancer immunotherapy.

## Introduction

Tumor cells can suppress immunity both systemically and in the tumor microenvironment^1^. Thus, initiating or boosting immune responses to tumors is the primary aim of current immunotherapies^2^. However, despite the promising clinical trials with vaccines^3^, adoptive T-cell transfer^4^, and check point inhibitor immunotherapies^5^, it is now being increasingly recognized that tumors develop additional immunosuppressive mechanisms countering the effective immunotherapies via inhibiting the tumoricidal effects of cytotoxic T lymphocytes^6,7^.

Tumor-associated macrophages (TAM)s derived from circulating monocytes^8^ are among the most abundant non-malignant cell types in the tumor microenvironment. TAMs are categorized as classically activated, tumor-suppressive M1 or alternatively activated, tumor-supportive M2 macrophages, which are capable of suppression of adaptive immunity by T cells as well as enhancement of angiogenesis, tumor cell invasion, and intravasation into blood vessels^9^. Despite numerous studies, the role of TAMs in the tumor microenvironment remains multifaceted. For instance, some studies have shown that high infiltration of TAMs correlates with poor prognosis in breast, gastric, oral, ovarian, bladder and thyroid cancer^10–14^, and blockade of colony-stimulating factor 1 receptor (CSF1R), essential for the recruitment, differentiation, and survival of TAMs, reduces the TAM infiltration and their immunosuppressive functions, which impairs tumor progression^15–17^. However, other studies, in particular on lung cancer, suggest that the role of TAMs is more complex. For instance, some studies on non-small cell lung carcinoma (NSCLC) demonstrated that there is no significant correlation of TAM densities with disease-specific survival^18^, while others showed that high TAM densities were associated with poor survival rate but not with TNM stages in human adenocarcinoma and squamous cell carcinoma lung cancer^19^. Interestingly, another study showed that M1 TAM density was an independent predictor of survival time but M2 TAM density was not significantly different between the long survival and short survival groups^20^.

Thus, given the multifaceted role of TAMs in lung cancer progression and in regulating anti-cancer immune response in particular, further studies identifying mechanisms that regulate TAM function in the tumor microenvironment are required.

In the present study, we demonstrate for the first time that caveolin-2 (Cav-2), a member of caveolin protein family that is largely dissimilar from its better known cousin, caveolin-1 in their amino acid sequence and function^21–25^, is critical for lung cancer growth through novel mechanisms involving TAMs and suppression of the anti-tumor immune response. Specifically, using a subcutaneously inoculated Lewis lung carcinoma (LLC) model of lung tumor growth in mice, we show a rapid increase in infiltration of M1-polarized and activated TAMs followed by CD4 and CD8 T cell infiltration and regression of tumors implanted into Cav-2 knockout (KO) mice. Transfer and co-injection of Cav-2 KO bone marrow (origin of TAMs) suppresses tumor growth and increases numbers of M1-polarized TAMs in wild type (WT) mice. Taken together, our data suggest that lung cancer cells use Cav-2 expressed in bone marrow-derived cell types including TAMs to promote tumor growth via inhibiting the anti-tumor immune response and that Cav-2 could be a potential target for cancer immunotherapy.

## Results

### Genetic deletion of Cav-2 in mice results in tumor rejection in transplantable syngeneic models of lung cancer progression

**Figure 1:**
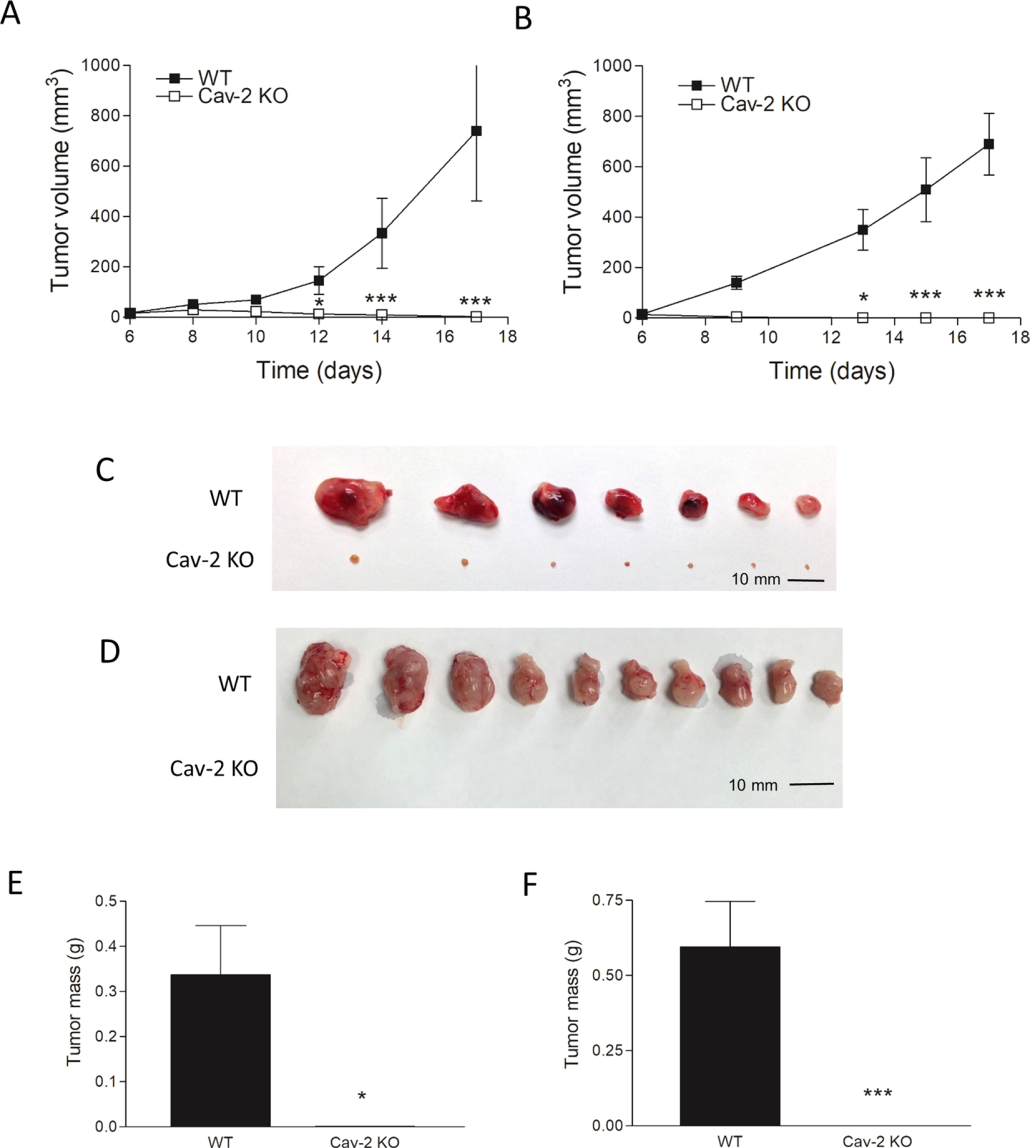
Subcutaneous growth of LLC and CMT 167 tumors in WT versus Cav-2 KO mice. (**A** and **B**) Graphical representation of LLC and CMT 167 tumor growth, respectively. LLC and CMT 167 cells were s.c. implanted into two flanks (10^6^/flank) of WT and Cav-2 KO mice. Tumor width and length were determined using a caliper, tumor volumes calculated according to the formula: Volume = 0.52 × (width)^2^ × (length). (**C** and **D**) Representative photographs of LLC and CMT 167 tumors extracted on day 17 of the experiment. (**E**,**F**) LLC and CMT 167 tumors extracted on day 17 (shown in **C** and **D**) were weighted and the average tumor mass ± SEM was calculated. Data are from distinct samples and expressed as the mean ± SEM. *p < 0.05 and ***p < 0.001 compared with WT by two-way ANOVA followed by Bonferroni post-test; n = 8 and 10 in A and B, respectively.

To examine the role of host-expressed Cav-2 in lung cancer progression, we used LLC^26^ and CMT 167^27^ as the two independent murine lung carcinoma cell lines derived in C57BL6 background. Initially, LLC cells were s.c. implanted into the flanks of WT and Cav-2 KO mice and tumor growth was determined as described in experimental procedures. As volume of LLC tumors rapidly and continuously increased in WT mice (Fig. 1A, closed squares), volume of LLC tumors in Cav-2 KO mice only increased till day 8, after which time point, LLC tumors started to shrink and regressed by day 17 (Fig. 1A, open squares). Next, we examined tumor growth using s.c. injected CMT 167 cells. Remarkably, even a more robust tumor growth inhibitory phenotype was observed in Cav-2 KO mice in the latter model of lung cancer growth compared to LLC model. Specifically, although compared to LLC, CMT 167 tumors implanted into WT mice grew more rapidly (Fig. 1B, closed squares), CMT 167 tumors implanted into Cav-2 KO mice were unable to grow past day 6 and completely regressed by day 13. (Fig. 1B, open squares). The remarkable reduction in LLC and CMT 167 tumor volume in Cav-2 KO mice on day 17 coincided with equally dramatic reduction in tumor size and mass determined upon surgical tumor removal followed by photographing (Fig. 1C,D) and weighing (Fig. 1E,F).

Taken together, our data suggest that host deficiency in Cav-2 causes tumor regression in two independent syngeneic models of lung cancer growth in immunocompetent mice involving s.c. injection of LLC and CMT 167 lung carcinoma cells. Due to a rapid regression of CMT 167 tumors in Cav-2 KO mice, which prevented us from obtaining cell numbers and tissue mass sufficient for further downstream applications, we have used s.c. implanted LLC cells as a model for mechanistic studies described below.

### Early stage LLC tumors from Cav-2 KO mice have increased macrophage as well as CD4 and CD8 T cell infiltration

**Figure 2:**
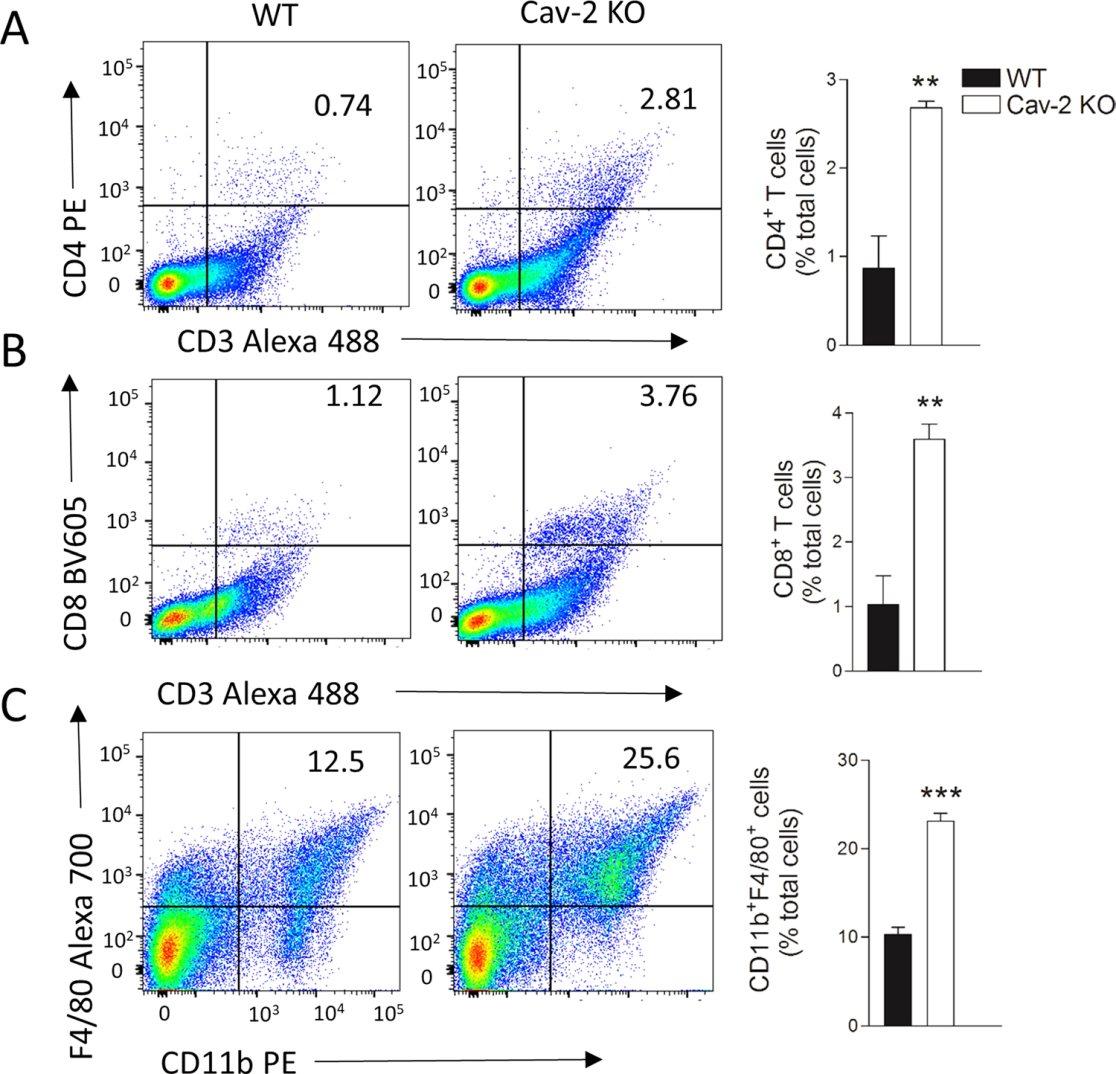
LLC tumor infiltrating CD4 and CD8 T cells as well as macrophages in WT versus Cav-2 KO mice analyzed by flow cytometry. Tumors were extracted at day 8 after s.c. implantation of LLC cells into flanks (10^6^/flank) of WT and Cav-2 KO mice and single cell suspensions were prepared and stained with fluorescence-conjugated antibodies for flow cytometry as described in Methods. (**A**–**C**) Flow cytometry analysis of single cell suspensions obtained from LLC tumors and stained for detecting CD4 T cells (CD3^+^CD4^+^), CD8 T cells (CD3^+^CD8^+^) and macrophages (CD11b^+^F4/80^+^), respectively. Left and central panels: representative dot plots with indicated percentages of T cells and macrophage populations within total cells in LLC tumors from WT and Cav-2 KO mice, respectively. Right panels: percentages of T cells and macrophages calculated based on the respective dot plots. All plots were gated on total live cells. Data are from distinct samples and presented as the mean ± SEM. **p < 0.01, ***p < 0.001 compared with WT by unpaired t-test; n = 4.

To investigate if the immune cell types may be involved in tumor rejection observed in Cav-2 KO immunocompetent mice, we used a multi-color flow cytometry approach. Specifically, tumors were extracted from WT and Cav-2 KO mice at day 8 after s.c. injection of LLC cells, the time point, at which tumors implanted into Cav-2 KO mice typically reached the maximum size before starting to regress (Fig. 1A, open squares) and their mass was still comparable with WT (Supplementary Fig. S1). Subsequently, single-cell solutions obtained from LLC tumors were stained using combinations of fluorochrome-conjugated antibodies against CD4^+^ and CD8^+^ T cells as well as macrophages, and the multi-color flow cytometry was performed in order to determine the frequency of these immune cell types within LLC tumors implanted into WT vs. Cav-2 KO mice. The complete gating strategy is shown in Supplementary Fig. 2. The results of these studies revealed a significant, ca. 3-fold increase in the numbers of both infiltrating CD4^+^ and CD8^+^ T cells in tumors extracted from Cav-2 KO mice (Fig. 2A,B). Moreover, there was a significant, ca. 2-fold increase in numbers of infiltrating F4/80^+^CD11b^+^ macrophages into tumors from Cav-2 KO mice (Fig. 2C). Taken together, these data suggest that the increased T cell and macrophage infiltration into LLC tumors may contribute to the anti-tumor phenotype observed in Cav-2 KO mice implanted with LLC cells.

### LLC tumors from Cav-2 KO mice have increased levels of macrophage-specific surface markers and M1-polarized macrophage-produced cytokines prior to increased mRNA levels of CD4 and CD8 T cell-specific markers

**Figure 3:**
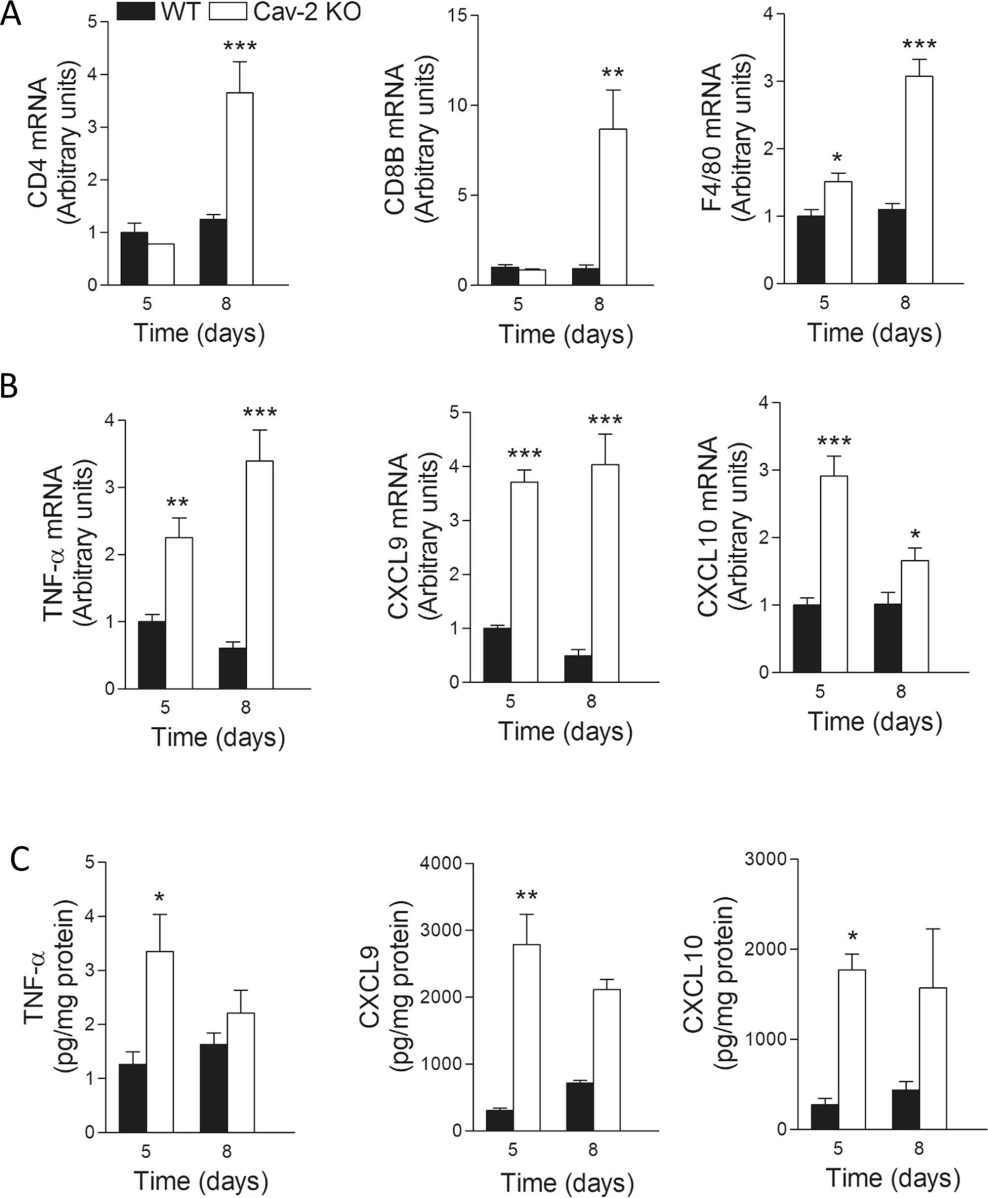
mRNA and protein levels for selected surface markers and cytokines in LLC tumor lysates from WT versus Cav-2 KO mice. (**A**,**B**) RNA was extracted from tumors 5 days and 8 days after s.c. implantation of LLC cells into WT and Cav-2 KO mice and mRNA levels were determined by RT-qPCR. The values are presented as the mean ± SEM. *p < 0.05; **p < 0.01; ***p < 0.001 vs. WT determined by unpaired t-test; n = 4. (**C**) Protein lysates were generated from tumors extracted from WT and Cav-2 KO mice 5 and 8 days after s.c. injection of LLC cells. Concentrations of each cytokine were determined using multiplex bead-based Discovery assay by Eve Technologies and normalized per mg of protein. Data are from distinct samples and presented as the mean ± SEM. *p < 0.05, ***p < 0.001 compared with WT mice by unpaired t-test; n = 4.

To examine which immune cell type(s) may increase earlier and consequently initiate the tumor rejection cascade in Cav-2 KO mice we have used the earliest palpable tumors extracted at day 5 after s.c. implantation of LLC but we were unable to recover cell numbers sufficient for reliable flow cytometric analysis. Therefore, we used RT-qPCR to determine mRNA levels of selected T cell- and macrophage-specific surface markers as an alternative approach. Specifically, RT-qPCR analysis demonstrated that mRNA levels of CD4 and CD8 T cell-specific markers increased in tumors from Cav-2 KO compared to WT mice on day 8 but not on day 5 after LLC cell implantation (Fig. 3A, left and central panels). Importantly, in contrast to the delayed increase in CD4 and CD8 T cell-specific markers, the increase of mRNA levels for a macrophage-specific marker, F4/80 started early at day 5 and further progressed at day 8 (Fig. 3A, right panel). The latter suggests that increased infiltration of macrophages into LLC tumors implanted to Cav-2 KO mice occurs earlier than of CD4 and CD8 T cells.

Since the early tumor-infiltrating macrophages are typically M1-polarized^9,28^, we hypothesized that the earliest palpable LLC tumors from Cav-2 KO mice would have increased levels of M1-polarized macrophage-produced cytokines. To test our hypothesis, we examined the levels of selected M1-polarized macrophage-produced cytokines by a combination of RT-qPCR and the multiplex bead-based cytokine assays in LLC tumors extracted at day 5 and day 8 after s.c. implantation into WT and Cav-2 KO mice. Specifically, the mRNA levels of TNF-α, CXCL9 and CXCL10 cytokines (known to be produced by M1-polarized macrophages) were elevated early at day 5 and stayed elevated at day 8 in LLC tumors from Cav-2 KO vs. WT mice (Fig. 3B). Moreover, consistent with the early increase in M1-polarized macrophage-produced cytokines detected by RT-qPCR, the normalized protein levels of TNF-α, CXCL9 and CXCL10 determined by the cytokine assay in LLC tumor lysates also revealed the early increase in Cav-2 KO compared to WT at day 5 (Fig. 3C). Overall, our RT-qPCR and cytokine assay data suggest increased infiltration of M1-like TAMs into LLC tumors implanted into Cav-2 KO mice, which likely initiates T cell infiltration and consequently results in tumor regression.

### Early stage LLC tumors from Cav-2 KO mice have increased M1-like TAM infiltration

**Figure 4:**
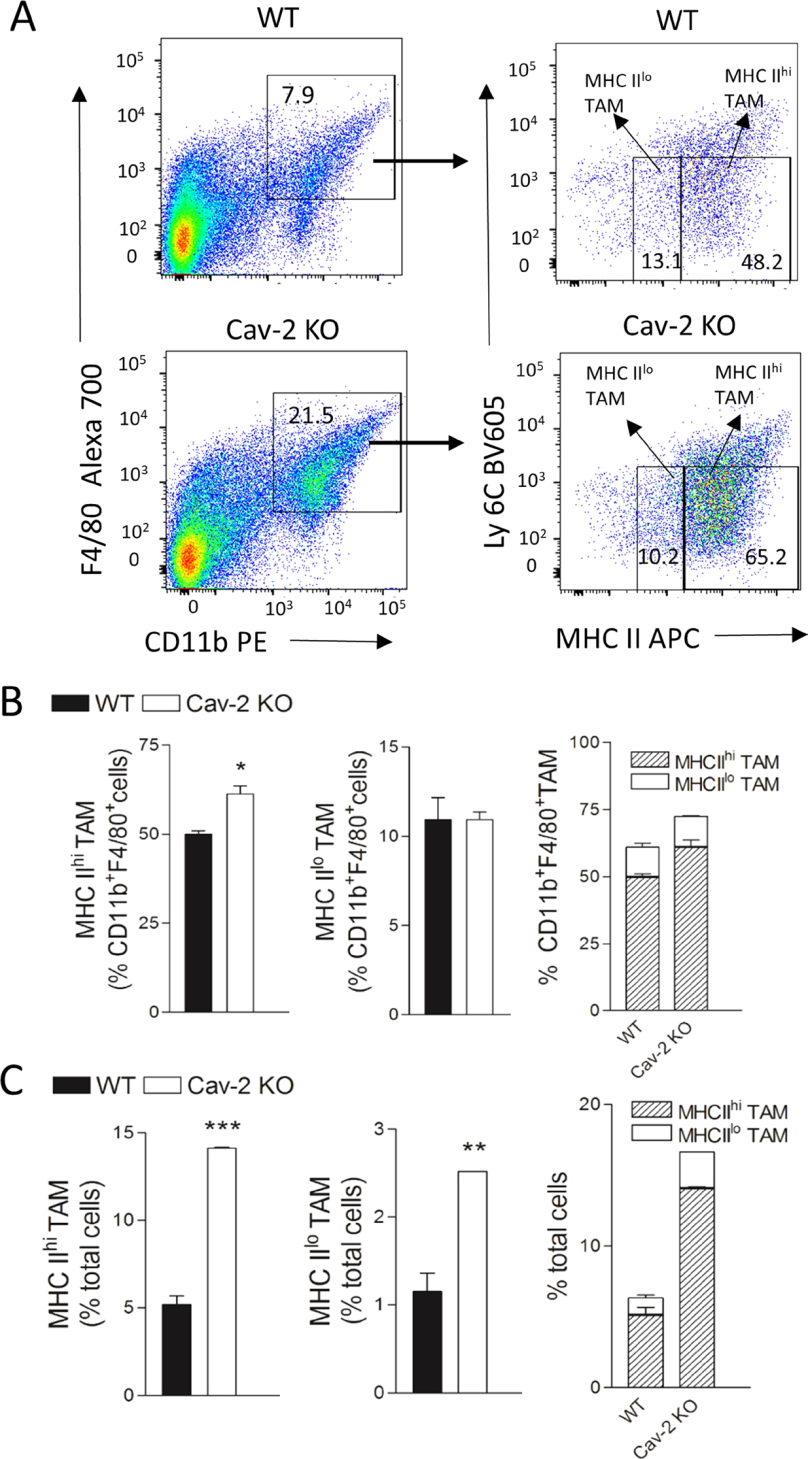
TAM subsets in subcutaneous LLC tumors from WT versus Cav-2 KO mice defined by flow cytometric analysis based on high and low MHC II expression levels. Tumors were extracted from WT and Cav-2 KO mice at day 8 after s.c. injection of LLC cells and single cell suspensions were prepared and stained with fluorescence-conjugated antibodies for flow cytometry as described in Methods. (**A**) Representative dot plots depicting gating strategy distinguishing MHC II^hi^ and MHC II^lo^ TAM subsets from LLC tumors implanted into WT (top panels) and Cav-2 KO mice (bottom panels). MHC II expressing cells, gated on CD11b^+^F4/80^+^ macrophages (left panels), were subsequently divided into Ly6C^lo^ MHC II^hi^ and Ly6C^lo^ MHC II^lo^ subsets, and defined as MHC II^hi^ TAM and MHC II^lo^ TAM, respectively (right panels). (**B**) Percentage of MHC II^hi^ TAM (left panel) and MHC II^lo^ TAM (central panel) within CD11b^+^F4/80^+^cells as well as combined percentages of MHC II^hi^ TAM versus MHC II^lo^ TAM within CD11b^+^F4/80^+^ cells (right panel) from WT and Cav-2 KO mice. (**C**) Percentage of MHC II^hi^ TAM (left panel) and MHC II^lo^ TAM (central panel) among total cells in LLC tumors  as well as combined percentage of MHC II^hi^ TAM versus MHC II^lo^ TAM among total cells (right panel) in LLC tumors from WT and Cav-2 KO mice. Data are from distinct samples and presented as the mean ± SEM. *p < 0.05, **p < 0.01; ***p < 0.001 compared with WT mice by unpaired t-test; n = 4.

To examine if LLC tumors implanted into Cav-2 KO mice may have higher numbers of infiltrating M1-polarized macrophages, we have used flow cytometry and a modified version of gating strategy previously reported^29^. A complete gating strategy is shown in Supplementary Fig. 2. Specifically, TAMs within single cell solution recovered from tumors extracted at day 8, initially gated as CD11b^+^F4/80^+^ cell populations (Fig. 4A, left panels), were analyzed based on the expression levels of Ly6C and MHC II markers. Consistent with the previously reported studies^29^, the Ly6C^lo^MHC II^hi^ and Ly6C^lo^ MHC II^lo^ TAMs were defined as MHC II^hi^ (M1-like) TAMs and MHC II^lo^ (M2-like) TAMs, respectively (Fig. 4A, right panels). As shown in Fig. 4B, the analysis of M1-like TAM and M2-like TAM subpopulations within CD11b^+^F4/80^+^ TAM populations clearly demonstrates a significant increase by ca. 1.23-fold in M1-like (Fig. 4B, left and right panel) but not M2-like (Fig. 4B; central and right panel) TAMs in LLC tumors from Cav-2 KO compared to WT mice. However, due to the increased total TAM infiltration (CD11b^+^F4/80^+^ cell), when the percentage of M1-like TAM and M2-like TAM is expressed relative to total cells in tumor (calculated by percentage of M1 or M2-like TAM multiplied by percentage of CD11b^+^F4/80^+^ cells), there is a nearly 3-fold increase in the percentage of M1-like TAMs and an over 2-fold increase of M2-like TAMs in LLC tumors from Cav-2 KO mice compared to WT mice (Fig. 4C, left and central panels). Finally, our results demonstrate that M1-like TAMs outnumber M2-like TAMs by 4.7- and 5.6-fold in LLC tumors from WT and Cav-2 KO mice, respectively (Fig. 4C, right panel). Taken together, our data demonstrate an increased TAM, predominantly M1-like TAM infiltration in LLC tumors from Cav-2 KO mice before they start to regress. Thus, the high M1-like TAM/M2-like TAM ratios in early stage tumors (observed in both WT and Cav-2 KO mice) may not be sufficient to suppress tumor growth without the overall increase in numbers of infiltrating M1-like TAMs (as observed in tumors from Cav-2 KO mice).

### Bone marrow transfer from Cav-2 KO mice inhibits LLC tumor growth in WT mice

**Figure 5:**
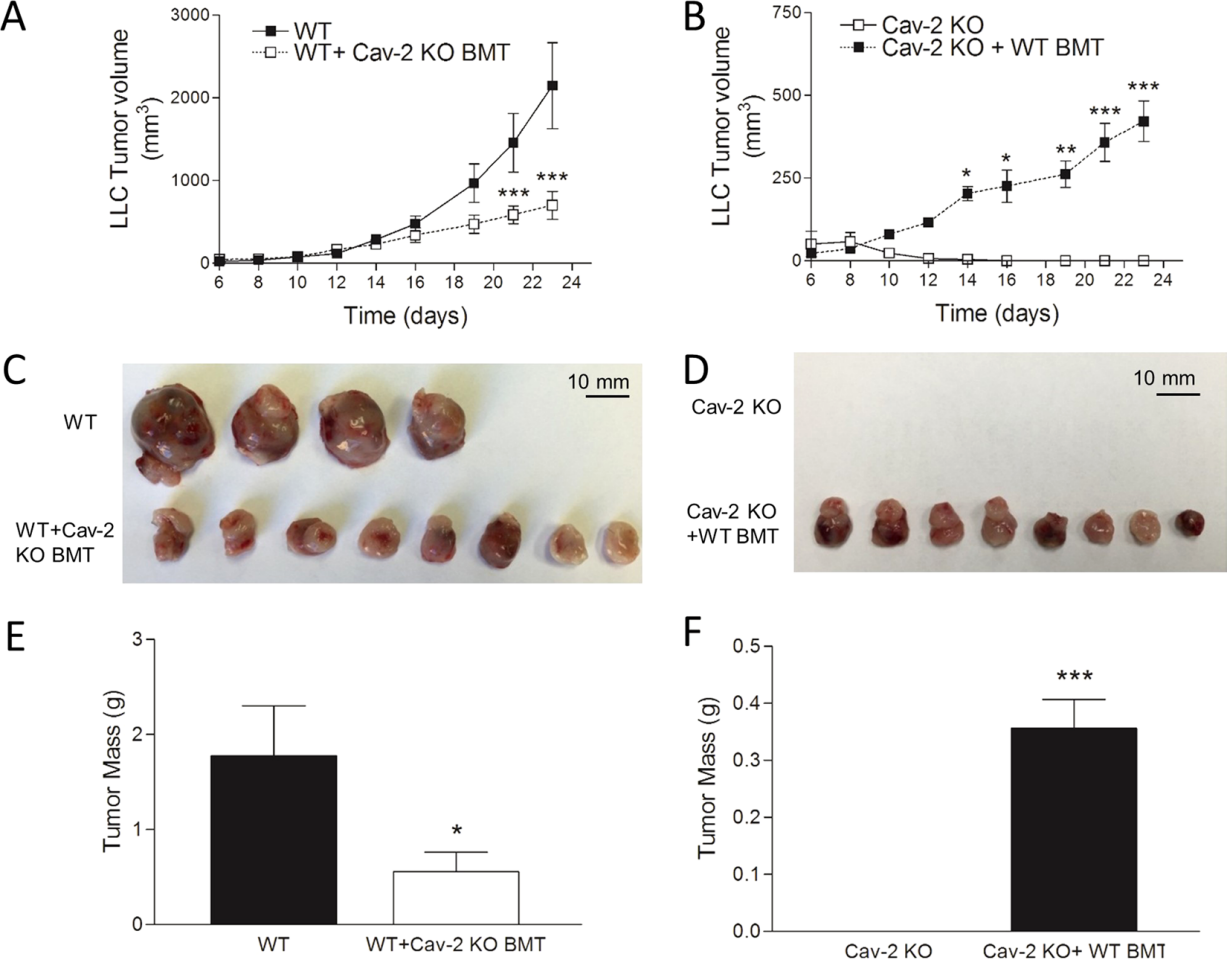
The effect of bone marrow transfer from Cav-2 KO and WT mice on LLC tumor growth. WT and Cav-2 KO mice irradiated with a lethal dose of 1000 Rads using an X-RAD 320 (X-RAY) received Cav-2 KO and WT bone marrow transfer (Cav-2 KO BMT and WT BMT, respectively) via intravenous (tail vein) injection of 5 × 10^6^ bone marrow cells isolated from murine femurs and tibia. 8 weeks after BMT, mice were s.c. injected with LLC cells (10^6^) into two flanks and tumor growth in WT + Cav-2 KO BMT (**A,C,E**) and Cav-2 KO + WT BMT (**B,D,F**) were compared to non-irradiated WT and Cav-2 KO control mice, respectively. A and B: graphical representations of LLC tumor growth in WT + Cav-2 KO BMT vs. WT and in Cav-2 KO + WT BMT vs. Cav-2 KO mice, respectively. Tumor width and length were determined using a caliper, tumor volumes were calculated according to the formula: Volume = 0.52 × (width)^2^ × (length), and data are  expressed as the mean ± SEM. *p < 0.05, **p < 0.01, and ***p < 0.001 compared with WT (**A**) and Cav-2 KO (**B**) by two-way ANOVA followed by Bonferroni post-test; (**C**,**D**) Representative photographs of LLC tumors extracted on day 23 of the experiment. (**E**,**F**) Tumors extracted on day 23 (shown in **C** and **D**) were weighted and the average tumor mass ± SEM was calculated. Data are from distinct samples and presented as the mean ± SEM. *p < 0.05 and ***p < 0.001 compared with WT (**E**) and Cav-2 KO (**F**) by unpaired t-test (n = 12).

TAMs originate from hematopoietic cells in bone marrow^30,31^. Thus, we hypothesized that Cav-2 KO bone marrow-derived hematopoietic cell types should be able to suppress LLC tumor growth in WT mice. To test the latter hypothesis, we performed bone marrow transfer experiments between Cav-2 KO and WT mice prior to s.c. injections of LLC cells as described in Methods. Consistent with our hypothesis, in WT mice receiving bone marrow from Cav-2 KO mice, the s.c. LLC tumor growth was significantly suppressed (Fig. 5A). Specifically, the fold of suppression gradually increased throughout the experimental timeframe and the appearance of significance started from day 21. The reduction of tumor volume on day 23 coincided with reduced tumor size and mass determined upon surgical tumor removal followed by photographing (Fig. 5C) and weighing, respectively. The average tumor mass was approximately 1.78 ± 0.52 g and 0.56 ± 0.20 g for WT mice without bone marrow transfer and for WT mice with Cav-2 KO bone marrow transfer, respectively (Fig. 5E), resulting in significant (p < 0.05) and nearly 3-fold reduction in tumor mass by Cav-2 KO bone marrow transfer (Fig. 5E).

To determine if WT bone marrow could potentially enable tumor growth in Cav-2 KO mice, we also performed reverse experiments, in which bone marrow from WT mice was transferred into Cav-2 KO mice. The results of these experiments demonstrate that WT bone marrow transfer to Cav-2 KO mice permits s.c. LLC tumor growth in Cav-2 KO mice (Fig. 5B). Specifically, instead of regression typically observed after day 8 in Cav-2 KO mice, LLC tumors were able to grow continuously in Cav-2 KO mice with WT bone marrow transfer and the tumor volume increased significantly starting on day 14 (Fig. 5B). On day 23, we were able to surgically extract large, aggressive growing tumors from Cav-2 KO mice receiving WT bone marrow transfer, with the mass about 0.36 ± 0.05 g, which was in stark contrast to Cav-2 KO mice that did not receive WT bone marrow transfer, and in which tumors completely regressed (Fig. 5D,F). Taken together, our data from bone marrow transfer experiments suggest that bone marrow-derived hematopoietic cell types from Cav-2 KO mice inhibit LLC tumor growth in WT mice, and that bone marrow-derived TAMs are possibly involved in this anti-LLC tumor phenotype.

### Cav-2 KO bone marrow co-injection increases M1-like TAM frequency within the early stage LLC tumors from WT mice

**Figure 6:**
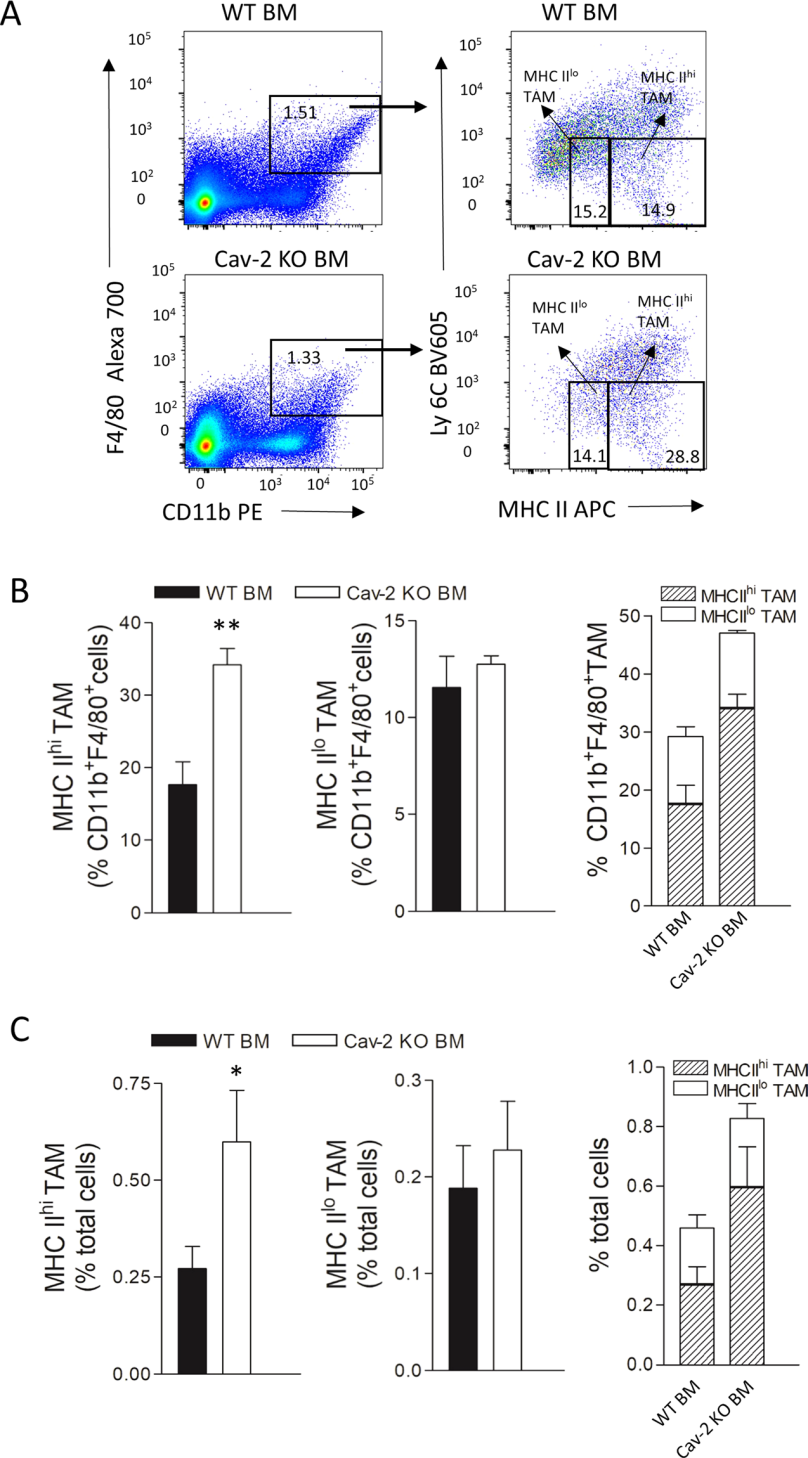
The effect of Cav-2 KO bone marrow cell co-injection on TAM subsets in LLC tumors. Freshly isolated bone marrow (BM) cells from WT vs. Cav-2 KO mice were co-injected s.c. with LLC (10^6^ cells) at 1:1 ratio in the lower back flanks of WT recipient mice as described in Methods. Tumors were extracted at day 8 and single cell suspensions were prepared and stained with fluorescence-conjugated antibodies for flow cytometry as described in Methods. (**A**) Gating strategy of MHC II^hi^ and MHC II^lo^ TAM subsets in LLC tumors from WT mice co-injected with WT BM (top panels) and Cav-2 KO BM (bottom panels). (**B**) Percentage of MHC II^hi^ TAM (left panel) and MHC II^lo^ TAM (central panel) within CD11b^+^F4/80^+^ cells  in LLC tumors as well as combined percentages of MHC II^hi^ TAM versus MHC II^lo^ TAM within CD11b^+^F4/80^+^ cells  (right panel). (**C**) Percentage of MHC II^hi^ TAM (left panel) and MHC II^lo^ TAM (central panel) among total cells in LLC tumors  as well as combined percentage of MHC II^hi^ TAM vs. MHC II^lo^ TAM among total cells in tumors  (right panel) from WT mice co-injected with WT vs. Cav-2 KO BM. Data are from distinct samples and presented as the mean ± SEM. *p < 0.05, **p < 0.01 compared with mice co-injected with WT BM by unpaired t-test; n = 4.

To provide a more direct evidence for the ability of Cav-2 KO bone marrow-derived hematopoietic cells to suppress tumor growth in WT mice and for involvement of M1-polarized TAMs, in addition to described previously bone marrow transfer, we performed experiments involving s.c. co-injections of Cav-2 KO vs. WT bone marrow with LLC cells into WT mice as described in Methods. Co-injection of Cav-2 KO bone marrow cells transiently suppressed LLC tumor growth in WT mice compared to their respective control counterparts co-injected with WT bone marrow cells (Supplementary Fig. S3). Next, we examined if changes in various TAM subsets is involved in initiating the tumor growth suppression by Cav-2 KO bone marrow cells. More specifically, we used flow cytometry to determine M1-like vs. M2-like TAM numbers from LLC tumors extracted at day 8, when the tumor volume was still comparable between mice co-injected with Cav-2 KO vs. WT bone marrow cells (Supplementary Fig. S3). Briefly, we used the same gating strategy as for TAM analysis in LLC tumors shown in Fig. 4. As shown in Fig. 6, the number of MHC II^hi^ (M1-like) TAMs was increased in LLC tumors co-injected with Cav-2 KO bone marrow cells relative to both CD11b^+^F4/80^+^ TAM populations (Fig. 6C; left panel) and to total cells in tumor (Fig. 6D; left panel). In contrast to MHC II^hi^ (M1-like), the number of MHC II^lo^ (M2-like) TAMs was not significantly different in tumors from mice co-injected with Cav-2 KO bone marrow compared to control mice co-injected with WT bone marrow cells (Fig. 6C,D; central panels). Taken together, our results suggest that Cav-2 KO bone marrow-derived hematopoietic cells are able to directly suppress LLC tumor growth and that the increased numbers of M1-like TAMs are involved.

## Discussion

Here, using immunocompetent Cav-2 KO mice as a unique model of a robust anti-tumor response to the implanted syngeneic lung carcinoma, we show for the first time that before starting to regress, the early stage tumors display an enhanced macrophage infiltration with M1-like phenotype followed by increased CD4 and CD8 T cell infiltration. The latter suggests that Cav-2 deficiency in the non-malignant cell types of the tumor microenvironment initiates a robust anti-tumor immune response to lung carcinoma mediated via increased infiltration and activation of M1-polarized macrophages, subsequent attraction of cytotoxic CD8 T cells into tumors, and tumor eradication in mice.

The robust anti-tumor phenotype in Cav-2 KO mice is evidenced by the results obtained using two independent syngeneic models of tumor growth in immunocompetent mice involving subcutaneous implantation with LLC and CMT 167 lung carcinoma cell lines. As the peak activity of cell-mediated anti-tumor immune response in mice occurs within 1 to 2 weeks^32,33^, a timeframe consistent with tumor rejection in Cav-2 KO mice, it is very likely that host deficiency in Cav-2 enhances the anti-tumor immune response. The slower tumor regression observed in Cav-2 KO mice s.c. implanted with LLC compared to CMT 167 proved to be critical for obtaining cell numbers sufficient for further analysis by flow cytometry as well as qRT-PCR and cytokine assays. Thus, we used s.c. LLC tumor growth model for subsequent mechanistic studies involving analysis of T cell and TAM subpopulations and activation.

Since the regression of s.c. implanted lung carcinoma tumors in Cav-2 KO mice started as early as after day 8, at which the tumor volume reached its maximum, these early stage tumors extracted at day 8 after LLC cell implantation became critical for our mechanistic studies examining the immune cell types using a multi-color flow cytometry approach. In addition, the volumes of these early stage LLC tumors from WT and Cav-2 KO mice were comparable, making the flow cytometry data with T cell and TAM numbers unaffected by differences in tumor size, which was not  the case at later time points due to continuous growth and regression of tumors implanted into WT and Cav-2 KO mice, respectively. Given well established role of cytotoxic CD8 T cells in the anti-tumor response, often leading to tumor regression, it was within our expectation that LLC tumors from Cav-2 KO mice were infiltrated with more CD8 T cells. Remarkably, in addition to CD4 and CD8 T cells, there was also increased infiltration of CD11b^+^F4/80^+^ TAMs into LLC tumors from Cav-2 KO mice. Historically, TAMs were believed to exist as mostly immunosuppressive, M2-polarized TAMs, supporting tumor growth and metastasis^34^. However, although the tumor microenvironment of later stage tumors contains large numbers of anti-inflammatory, M2-polarized macrophages promoting tumor growth and metastasis, this is not the case in the earlier stages of tumor development and growth^28^. For instance, it was proposed that during various stages of tumor progression, there is a gradual switch in TAM polarization from M1 to M2^35,36^, and early in carcinogenesis, M1 TAMs may contribute to tumor elimination^36^. Therefore, to test if TAMs in LLC tumors from Cav-2 KO mice have M1-like phenotype and if increase in M1-like TAM infiltration could possibly occur prior to CD4 and CD8 T cell infiltration, we went back to day 5, the earliest time point, at which the LLC tumors could be palpable and measurable in both WT and Cav-2 KO mice. However, due to very limited tumor volume at day 5, we were unable to recover cell numbers that were sufficient for flow cytometry. Thus, we employed a combination of RT-qPCR and cytokine assays as the alternate approaches. Consistent with our initial hypothesis, we have observed increased mRNA levels for general macrophage-specific marker, F4/80 and for M1-polarized macrophage-produced cytokines: TNF-α, CXCL9, CXCL10 in LLC tumors from Cav-2 KO mice compared to WT at both day 5 and day 8. Moreover, the results of a cytokine assay demonstrated increased production of TNF-α, CXCL9 and CXCL10 in tumors from Cav-2 KO mice  at the earliest time point after LLC injection and thus independently corroborated our findings obtained using RT-qPCR assay. Among others, the anti-tumor effects of CXCL10 and CXCL9 have been shown to be mediated through the recruitment of activated CD4 and CD8 T cells^37^ as well as through their inhibitory effects on tumor vasculature^38^. As for the earlier, our data with increased levels of CXCL9 and CXCL10 in the earliest stage tumors (at day 5 after implantation) is consistent with subsequently increased infiltration with CD4 and CD8 T cells in the early stage tumors (8 days after implantation). Conversely, consistent with the angiostatic effects of CXCL9 and CXCL10 by binding to their receptor CXCR3^38^, we earlier reported reduced angiogenesis in LLC tumors from Cav-2 KO mice as early as day 6 after tumor cell implantation^39^. Thus, our new data suggests that previously reported inhibition of tumor angiogenesis is a secondary effect caused by earlier infiltration with M1-polarized macrophages producing increased CXCL9 and CXCL10. In contrast to the previously discussed M1-polarized macrophage-specific or M1 macrophage-associated markers and cytokines, the mRNA levels for T cell-specific surface marker proteins, CD4 and CD8 were elevated only at day 8 but not at day 5, indicating that early increased M1-like macrophage infiltration into tumors implanted in Cav-2 KO mice may induce subsequent CD4 and CD8 T cell infiltration. In addition, the enhanced antigen presentation and T cell activation by M1-like TAMs and dendritic cells could possibly further contribute to increased CD4 and CD8 T cell infiltration, activation and ultimate tumor eradication observed in Cav-2 KO mice. Future studies will be required to establish the more exact mechanisms via which loss of Cav-2 in the host leads to tumor regression. However, based on the experimental evidence in this study, we believe that loss of Cav-2 in TAMs rather than in CD4 and CD8 T cells could initiate the anti-tumor immune response involving a combination of tumoricidal effects of M1-polarized TAMs and cytotoxic CD8 T cells leading to lung carcinoma tumor regression observed in Cav-2 KO mice.

It is important to note that the results of our studies using flow cytometry and gating strategy distinguishing (MHC II^hi^) M1-like TAM and (MHC II^lo^) M2-like TAM^29^ in the early stage LLC tumors clearly suggest increased percentage of M1-like TAM but not M2-like TAM within CD11b^+^F4/80^+^ TAMs in tumors from Cav-2 KO mice. Moreover, the analyses of M1- vs. M2-like TAM subpopulations relative to total cells within the tumor demonstrates a greater increase in M1- compared to M2-like TAMs in the early stage tumors from Cav-2 KO mice, suggesting that considerably higher numbers of M1-like TAMs appear to be critical for tipping the balance from pro-tumor to anti-tumor microenvironment and regression of lung carcinoma tumors in Cav-2 KO mice. Interestingly, although the ratios of M1-like to M2-like TAMs are still considerably high in the tumor microenvironment of WT mice, tumors continue growing, which may be due to insufficient numbers of infiltrating M1-polarized TAMs in WT compared to Cav-2 KO mice. Taken together, our findings suggest that both the high M1-like to M2-like TAM ratio and the increased numbers of infiltrating M1-like TAMs are required to initiate the anti-tumor immune response in Cav-2 KO mice. Based on these results, we suggest that not only polarization to M1 phenotype but also concurrent increase in TAM infiltration, as opposed to eliminating TAMs using function-blocking antibodies and small molecule-based inhibitors, should ultimately prove most effective for successful TAM-centered immunotherapy. For instance, attempts were made to influence TAM phenotype using attenuated Listeria monocytogenes to repolarize TAMs to the pro-inflammatory phenotype^40,41^, but the benefit is still limited. In another study, using s.c. LLC model, Liu *et al*.^42^ reported that β-glucan, a potent immunomodulator, is able to convert immunosuppressive M2 TAMs into the M1-polarized macrophages resulting in suppression of LLC tumor growth. However, the suppression is a lot less robust compared to the suppression in our Cav-2 KO mice. The same study also demonstrated that depletion of macrophages with Clodronate significantly reduces β-glucan-mediated therapeutic efficacy, which partially support our data that if we provide more supply rather than depletion of macrophages combined with activated M1 phenotype, the tumors might be eliminated (as it happened in Cav-2 KO mice implanted with lung carcinoma cells).

TAMs originate from hematopoietic cells in bone marrow^30,31^. Bone marrow transfer to irradiated mice is a powerful approach to investigate immune cell function. Although in addition to TAM, bone marrow is the origin of several other immune cell types, we already demonstrated that Cav-2 deficient TAMs rather than CD4 and CD8 T cells initiate anti-tumor cascade. As we expected, bone marrow transferred from Cav-2 KO mice significantly suppressed LLC growth in WT mice and the replacement of WT bone marrow on Cav-2 KO mice induced LLC growth, suggesting that Cav-2 deficiency and Cav-2 expression in bone marrow-derived cell types is important for suppressing and promoting tumor growth, respectively. The mechanism via which bone marrow-derived cell-expressed Cav-2 promotes tumor growth remains to be examined but could potentially involve suppression of M1 TAMs implied by our flow cytometry data demonstrating increased M1-like TAM frequency in the early stage LLC tumors with co-injected Cav-2 KO bone marrow cells. Thus, additional studies examining if Cav-2 expressed in bone marrow-derived cell types regulates M1-like TAM expansion and/or recruitment are clearly warranted. In addition, further studies will be necessary to prove the direct relationship between Cav-2 and TAM in regulating tumor growth and anti-tumor immune response. For instance, it will be important to examine if macrophage-expressed Cav-2 will directly regulate M1-like TAM expansion, polarization and/or recruitment. Comprehensive studies examining Cav-2 expression spectrum among different tumor-infiltrating bone marrow-derived cell types including (but not limited to) M1-like vs. M2-like TAMs, monocytes, polymorphonuclear leukocytes, NKT and NK cells during earlier vs. later stages of tumor growth will undoubtedly shed more light into better understanding of the mechanisms via which Cav-2 regulates tumor growth and anti-tumor immune response.

Consistent with the notion that basic bioscience research should aimed at clinical application, the application of cancer immunology is cancer immunotherapy. However, although current immunotherapies such as checkpoint inhibitions and vaccines aimed at activation of cytotoxic T cells, have made a considerable progress, the response rate is not as high as expected. Recently, more studies have been focused on immune suppressive cell types such as TAMs, including TAM depletion or re-polarization to M1 phenotype, but the response is still limited. Here, using our unique anti-tumor model involving Cav-2 deficient mice, we demonstrate that the combination of M1 phenotype and augmented TAM infiltration is likely required to initiate the LLC tumor eradication. We believe that this discovery will not only contribute to better understanding of cancer immunology but will also provide a basic theory for designing more robust cancer immunotherapies, presumably involving strategies combining enhanced TAM infiltration and TAM polarization to M1 phenotype.

## Methods

### Cell lines

LLC (ATCC) and CMT 167 (a gift from Dr. Raphael Nemenoff (University of Colorado)) murine lung carcinoma cell lines were cultured and authenticated as described previously^39^. Briefly, cells were cultured in DMEM containing 10% FBS, 1% L-glutamine and 100 UI/mL of penicillin plus streptomycin in a humidified chamber at 37 °C under 5% CO2. Both cell lines were regularly authenticated according to the guidelines provided by the ATCC based on morphology (rounded–loosely attached or floating for LLCs and epithelial-like, closely packed sheets at confluence for CMT 167), viability, recovery, and growth, most recently confirmed one month before use.

### Mice

6- to 8-week-old Cav-2 KO and WT littermate mice in C57BL/6N were used for all experiments according to the protocol approved by the University of Missouri Animal Care and Use Committee as described previously^39^. Cav-2 KO mice were generated in C57BL/6N background, originating from Charles River Laboratories, with the assistance of Mouse Biology Program (UC Davis, CA) by deletion of entire exon 2 and a 50 portion of exon 3 as described previously^39^.

### Tumor cell implantation and growth

Tumor growth experiments were performed as described previously^39^. Briefly, LLC or CMT 167 (10^6^ cells in 100 mL PBS) were injected s.c. in the lower back flanks of 6- to 8-week-old Cav-2 KO and WT littermate mice. When tumors became palpable (typically on day 5–6 after implantation), LLC and CMT 167 tumor growth was monitored every other day by measuring the length and width of the tumor using a caliper. Tumor volume was calculated using the following formula: Volume = 0.52 × (width)^2^ × (length). In addition, at the end of the experiments (day 17), tumors were removed and tumor mass was determined by weighing.

### Tumor cells preparation and multicolor staining analyses by flow cytometry

Extracted tumors were minced completely (1–3 mm cubes) by using scalpels in two drops of dissociation solution (1 mg/ml collagenase D (11088858001 Roche, Indianapolis, IN) and 1 mg/ml DNase I (10104159001 Roche) in RPMI 1640) on ice. Tumor pieces were further digested for 45 min with dissociation solution (about 1 g tumor in 10 ml dissociation solution) at 37 °C water bath with manually periodical shaking. The digested tissue suspension was aspirated into a 20 ml syringe with 14 g cannula attached and clumps were triturated 15 times and filtered through 40 um strainer to get the single cell suspension  followed by staining with fluorescent antibodies from Biolegend: PE CD4 (GK1.5, 100407), Brilliant Violet 605 CD8a (53–6.7, 100744), PE CD11b (M1/70, 101207), Alexa Fluor 700 F4/80 (BM8, 123130), Alexa Fluor 488 CD3 (17A2, 100210), APC MHC II (M5/114.15.2, 107613). Stained cells were analyzed using BD LSRFortessa X-20 flow cytometer. Data were collected with BD FACSDiva v 8.0.1 software and were analyzed using FlowJo v 10 software.

### RNA isolation and analysis of specific gene expression by quantitative real time PCR

Quantitative real time PCR (qRT-PCR) – qRT-PCR was performed as  described previously^43,44^ with minor modifications. Briefly, total RNA was isolated from LLC tumors using TRI reagent (Sigma). 1ug of isolated RNA was then reverse transcribed into cDNA using iScript™ cDNA Synthesis kit (Bio-Rad). Relative mRNA expression levels were determined by qRT-PCR. 18S ribosomal RNA (18S rRNA) was used as the housekeeping gene, and gene expression was measured using Bullseye EvaGreen qPCR Mix (Midwest Scientific) with the BIO-RAD CFX 96 Touch RT-PCR Thermal Cycler. Data were collected with BIO-RAD CFX Manager 3.1 software. The following primers were used for the amplification of mouse: 18S rRNA (RefSeq ID: NM_011296): forward, 5′-GTGATCCCTGAGAAGTTCCAG-3′ and reverse, 5′-TCGATGTCTGCTTTCCTCAAC-3′; CD4 (RefSeq ID: NM_013488): forward, 5′-GTTCGGCATGACACTCTCAG-3′ and reverse, 5′-CCTTCTCTGCCTTCCACATC-3′; CD8b (RefSeq ID: NM_009858): forward, 5′-TGGCCGTCTACTTTTACTGTG-3′ and reverse, 5′-GGCGCTGATCATTTGTGAAAC-3′; CD11b (Itgam, RefSeq ID: NM_001082960): forward, 5′-CATCCCATGACCTTCCAAGAG-3′ and reverse, 5′-GTGCTGTAGTCACACTGGTAG-3′; F4/80 (Emr1; RefSeq ID: NM_010130): forward, 5′-ACCACAATACCTACATGCACC-3′ and reverse, 5′-AAGCAGGCGAGGAAAAGATAG-3′; Tumor necrosis factor alpha (TNF-α; RefSeq ID: NM_013693): forward, 5′-ATCTGAGTGTGAGGGTCTGGGC -3′ and reverse, 5′-AGACCCTCACACTCAGATCA-3′; C-X-C motif chemokine 9 (Cxcl9, RefSeq ID: NM_008599): forward, 5′-AGTCCGCTGTTCTTTTCCTC-3′ and reverse, 5′-TGAGGTCTTTGAGGGATTTGTAG-3′; C-X-C motif chemokine 10 (Cxcl10, RefSeq ID: NM_021274): forward, 5′-TCAGCACCATGAACCCAAG-3′ and reverse, 5′-CTATGGCCCTCATTCTCACTG-3′. Thermal conditions were 15 minutes at 95 °C, and 40 cycles of 10 seconds at 95 °C, 30 seconds at 52 °C and 30 seconds at 72 °C. Values are calculated based on the amount of target mRNA normalized to the endogenous reference 18S rRNA mRNA based on the following equation: 2^−ΔΔCt^. Data are expressed as mean ± SEM of 2–3 samples in 2 replications (n = 4–6) from one representative out of 3 total experiments.

### Tumor lysate preparation and cytokine assay

Tumor cell lysates were prepared as described previously^39^. Specifically, LLC tumor tissue samples were extracted from mice and snap-frozen in liquid nitrogen followed by extraction with tissue grinder and lysis in a RIPA lysis buffer containing: 50 mmol/L Tris HCl, 0.1 mmol/L EGTA, 0.1 mmol/L EDTA, 100 mmol/L leupeptin, 1 mmol/L phenylmethylsulfonyl fluoride, 1% (v/v) NP-40, 0.1% SDS, and 0.5% deoxycholic acid; pH 7.4, homogenized, and centrifuged for 10 minutes at 14,000 rpm and at 4 °C. Supernatants were analyzed using multiplex bead-based Discovery assay by Eve Technologies, Canada.

### Bone marrow transplantation

Six-week-old C57BL/6N WT and Cav-2 KO recipient mice were maintained on sulfamethoxazole and trimethoprim (TMS; 1 mg/ml, STI Pharms, LLC. NDC 54879-007-16) in drinking water (acidic water, pH = 2.6) one week before irradiation and 2 weeks after bone marrow cell injection. The acidic water without antibiotics was provided for the rest of their lives. A single dose of 1000R from X-Rad 320 irradiator was used to irradiate recipient mice. Four hours later, 5 × 10^6^ bone marrow cells isolated from six-week-old donor mice were injected via tail vein into each recipient mouse. Eight weeks after bone marrow transplantation, LLC (10^6^ cells in 100 mL PBS) was injected s.c. in the lower back flanks of recipient mice. Tumor growth was monitored every other day using a caliper from day 6 and tumors were removed at day 23 for photographing and weighing.

### Bone marrow and LLC cell co-injection

Freshly isolated bone marrow cells isolated from the femurs and tibia of six-week-old WT and Cav-2 KO mice were co-injected s.c. with LLC (10^6^ cells) at 1:1 ratio in the lower back flanks of recipient WT mice. Tumor growth was monitored every other day using a caliper from day 6 until day 18. In a separate experiment, LLC tumors from mice co-injected with WT vs. Cav-2 KO bone marrow were extracted at day 8 and processed for the analysis of flow cytometry.

### Statistical analysis

Data analysis was performed as described previously^39^. Specifically, for the experiments involving time-dependent growth of LLC and CMT 167 tumor grafts, the data are expressed as mean ± SEM (n = 6–8). To determine the statistical significance, the two-way ANOVA was used followed by the Bonferroni post-test. For all other experiments the Student t test was performed. Differences were considered statistically significant *p< 0.05.

## Supplementary information

Supplementary Information

## Data Availability

The authors declare that any data supporting the findings described in this study are available from the corresponding author upon reasonable request.
