# Supplementary material for: Caveolin-2 deficiency induces a rapid anti-tumor immune response prior to regression of implanted murine lung carcinoma tumors
Source: Sci Rep. 2019 Dec 12;9:18970. doi: 10.1038/s41598-019-55368-4 (PMC6908574; doi:10.1038/s41598-019-55368-4)
Supplement: Supplementary file 1 — Supplementary Information [file 41598_2019_55368_MOESM1_ESM.pdf]

Supplementary Information for manuscript "Caveolin-2 deficiency induces a rapid anti-tumor immune response prior to regression of implanted murine lung carcinoma tumors" by Yajun Liu, Xiaoqiang Qi, Guangfu Li and Grzegorz Sowa

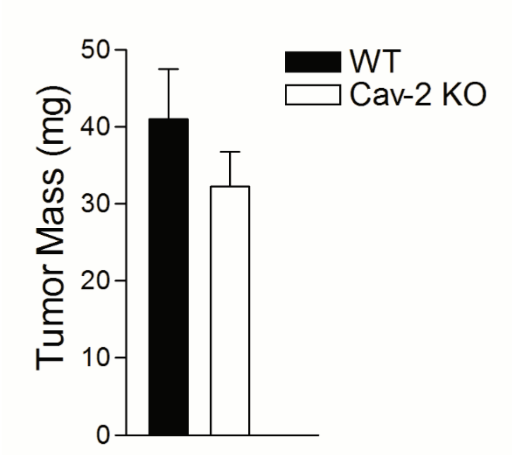
WT
Cav-2 KO
Tumor Mass (mg)
50
40
30
20
10
0

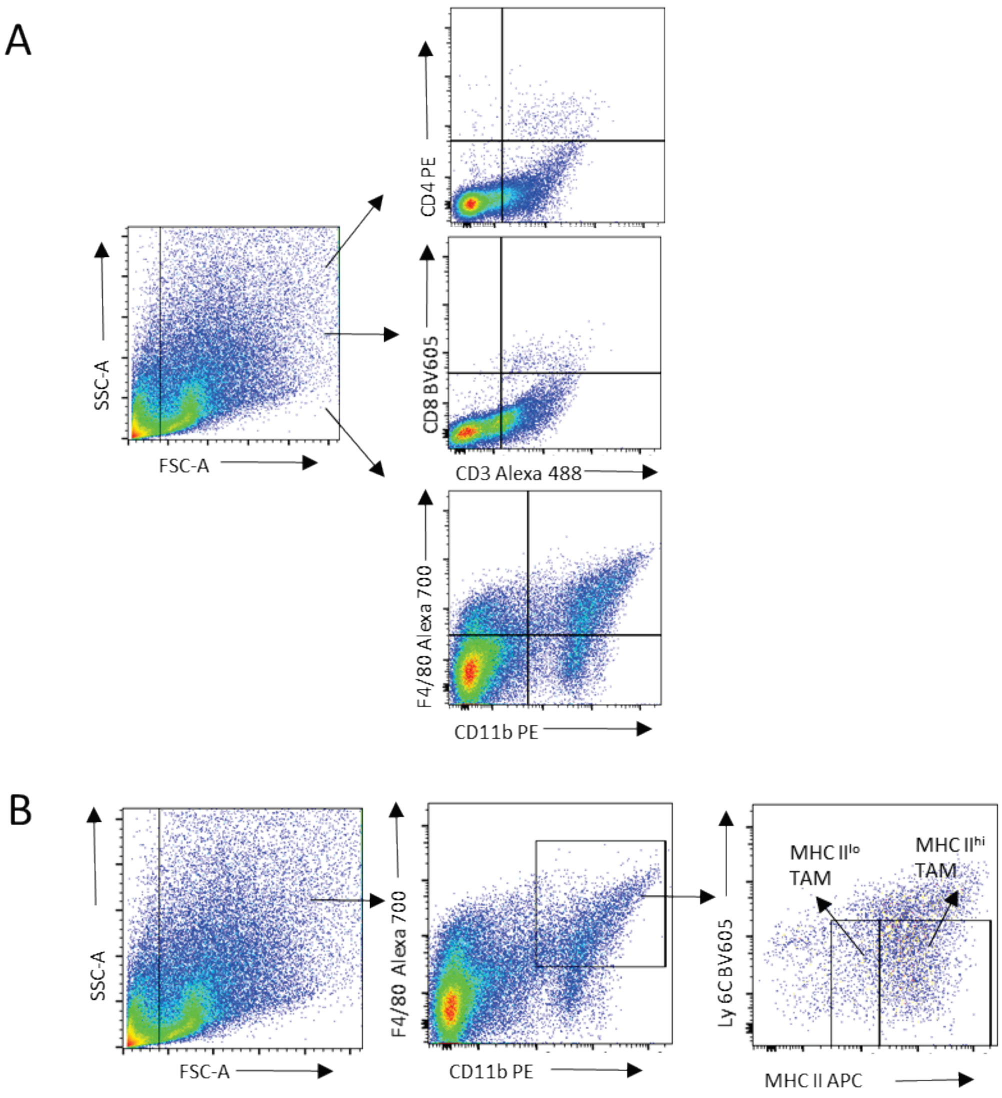
A
SSC-A
FSC-A
CD4 PE
CD8 BV605
CD3 Alexa 488
F4/80 Alexa 700
CD11b PE
B
SSC-A
FSC-A
F4/80 Alexa 700
CD11b PE
Ly 6C BV605
MHC II APC
MHC II lo TAM
MHC II hi TAM

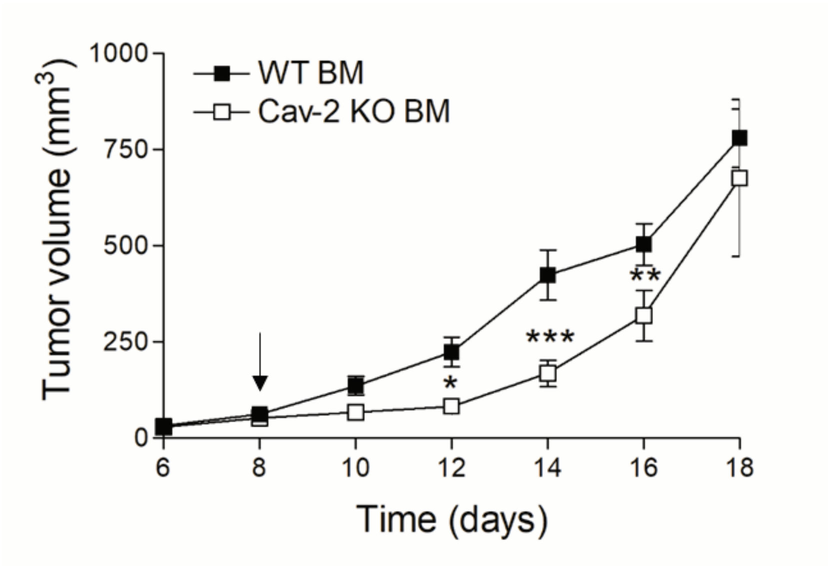
WT BM
Cav-2 KO BM
Tumor volume (mm3)
1000
750
500
250
0
6
8
10
12
14
16
18
Time (days)
*
***
**

## Supplementary Figure legends

**Supplementary Figure S1. Comparison of the early stage LLC tumor mass from WT versus Cav-2 KO mice.** Tumors extracted at day 8 after s.c. implantation of LLC cells into flanks ($10^6$/flank) of WT and Cav-2 KO mice were weighted and the average tumor mass ± SEM was calculated from distinct samples. The data analysis by the unpaired t-test revealed that there was no statistically significant difference in tumor mass between WT and Cav-2 KO mice (n = 6).

**Supplementary Figure S2. Gating strategy for flow cytometry analysis.** Tumors were extracted from WT and Cav-2 KO mice at day 8 after s.c. injection of LLC cells and single cell suspensions were prepared and stained with fluorescence-conjugated antibodies for flow cytometry as described in Methods. A. Gating strategy for flow cytometry analysis of tumor infiltrating CD4 T cells, CD8 T cells and $CD11b^+F4/80^+$ macrophages. B. Gating strategy of flow cytometry analysis of tumor infiltrating MHC II expressing macrophages.

**Supplementary Figure S3. The effect of Cav-2 KO bone marrow cell co-injection on LLC tumor growth in WT mice.** Freshly isolated bone marrow (BM) cells from WT vs. Cav-2 KO mice were co-injected s.c. with LLC cells ($10^6$ cells) at 1:1 ratio in the lower back flanks of WT recipient mice. Tumor growth was monitored every other day using a caliper from day 6 until day 18. Data are from distinct samples and expressed as the mean ± SEM. * $p < 0.05$, ** $p<0.01$, *** $p <0.001$ compared with LLC+ WT BM by two-way ANOVA followed by Bonferroni post-test; n = 8 - 10. Arrow depicts early stage tumors at day 8 after co-injection, which were of

similar size to those used for flow cytometric assessment of M1-like TAM numbers (shown in Fig. 6).
